# Sequence-based detection of mutations in cadherin 1 to determine the prevalence of germline mutations in patients with invasive lobular carcinoma of the breast

**DOI:** 10.1186/1897-4287-12-17

**Published:** 2014-07-19

**Authors:** Allyson L Valente, Seth Rummel, Craig D Shriver, Rachel E Ellsworth

**Affiliations:** 1 Clinical Breast Care Project, Windber Research Institute, 620 Seventh Street, Windber, PA, USA; 2 Clinical Breast Care Project, Walter Reed National Military Medical Center, 8901 Wisconsin Ave, Bethesda, MD, USA; 3 Clinical Breast Care Project, Henry M. Jackson Foundation for the Advancement of Military Medicine, 620 Seventh Street, Windber, PA, USA

**Keywords:** Breast cancer, ILCA, CDH1

## Abstract

**Background:**

Loss of cadherin 1 (CDH1) expression, which is normally involved in cell adhesion and maintenance of tissue architecture, is a hallmark of invasive lobular carcinoma (ILCA). Because hereditary cancers may require different risk reduction, counseling and treatment options than sporadic cancer, it is critical to determine the prevalence of germline CDH1 mutations in patients with ILCA.

**Methods:**

All patients with ILCA (n = 100) previously enrolled in the Clinical Breast Care Project were identified. Genomic DNA was isolated from peripheral blood samples and DNA variants were detected for each exon of CDH1 using high-resolution melting technology followed by direct sequencing.

**Results:**

Within the 100 samples screened, four nonsynonymous variants were detected: A592T in one Hispanic patient, A617T in two patients, both African American, P825L in a Causasian patient whose grandmother had stomach cancer, and G879S in a Caucasian patient. Further evaluation of A617T in an additional 165 African American patients found that 11 patients, none with ILCA, carried this variant including one patient who was homozygous for the variant.

**Conclusions:**

CDH1 mutations are infrequent in patients with ILCA, and the variants that were detected have been classified as non-pathogenic. These data suggest that ILCA does not have a significant hereditary component and do not support CDH1 gene mutation testing in patients with ILCA.

## Background

Breast cancer represents a group of diseases, with different histological, molecular and clinical characteristics, including tumor growth patterns, with at least 17 different histological types currently described [1]. Invasive lobular carcinoma (ILCA), the second most common histological type, accounts for 8-14% of all breast cancers [2,3]. ILCA is histologically characterized by a loss of cellular adhesion, resulting in growth of single cells or cells arranged in a single file or “Indian lines” [3,4]. ILCA is often bilateral, multifocal and multicentric, and is more difficult to detect by palpation or mammography [5,6]. Compared to invasive ductal carcinoma (IDCA), ILCA is more often diagnosed at a later age, more likely to be hormone receptor positive, larger, diploid, and HER2, p53 and EGFR negative. ILCA has a distinctive pattern of metastasis, with a propensity to metastasize to the peritoneum, gastrointestinal tract and ovaries. Despite its favorable characteristics, disease-free and overall survival did not differ between ILCA and IDCA [7].

One of the hallmarks of ILCA is the loss or altered expression of cadherin 1 (CDH1, GenBank: AB025105.1). CDH1 is a member of the classical/type I cadherin family that is involved in maintaining cellular adhesion. CDH1 interacts with members of the catenin family; this complex then links with actin molecules to provide cellular adhesion [8]. Altered expression of CHD1 explains the discohesive phenotype characteristic of not only ILCA but also hereditary diffuse gastric cancer (HDGC). In fact, ILCA is a frequent finding in HDGC families, with a 39% lifetime risk of developing ILCA in HDGC families with CDH1 germline mutations [9].

The frequency of CDH1 germline mutations in families with HDGC is 25-30% [10]. Determination of the frequency of CDH1 mutations in patients with ILCA is crucial; clinically, the diffuse nature of ILCA makes early detection more challenging [5] and increased risk for bilateral disease impacts the choice of surgical approaches. In addition, presence of hereditary CDH1 mutations will impact risk assessment, family counseling and screening, for both breast and stomach malignancies. To this end, CDH1 was screened for germline mutations in a population of patients with ILCA.

## Methods

All patients included in this study were enrolled in the Clinical Breast Care Project (CBCP) between 2001 and 2010. Blood samples were collected with approval from the Walter Reed National Military Medical Center Human Use Committee and Institutional Review Board. All subjects voluntarily agreed to participate and gave written informed consent. Clinical information, including family cancer history, was obtained for all CBCP participants using questionnaires designed by and administered under the auspices of the CBCP.

The CBCP database was queried to identify all female patients diagnosed with ILCA. Family history of cancers was self-described by the patients and included data for both primary and secondary relatives. Diagnosis of every specimen was performed by a dedicated breast pathologist from hematoxylin and eosin (H&E) stained slides; staging was performed using guidelines defined by the AJCC *Cancer Staging Manual* seventh edition [11]. In addition, to determine the frequency of the A617T variant in African American women, all female African Americans with invasive breast cancers such as IDCA and tumors with mixed IDCA and ILCA features were identified.

Genomic DNA was isolated from blood clots using the GentraClotspin and Puregene DNA purification kits (Qiagen, Valencia, CA). Polymerase chain reaction (PCR) primers were designed to flank CDH1 exons 1–15 and the coding region of exon 16 (Table 1). PCR reactions were performed on 25 ng genomic DNA using Lightscanner Master Mix (BioFire Defense, Salt Lake City, UT) and analyzed on the LightScanner System following manufacturer’s protocols. For those samples with a variant detected by the LightScanner, PCR products were purified using ExoSAP-IT For PCR Product Clean-up (Affymetrix, Santa Clara, CA), and sequenced using BigDye terminator v 3.1 cycle sequencing kits (Life Technologies, Inc, Carlsbad, CA). The resulting products were sequenced on a 3730xl DNA Analyzer (Life Technologies, Inc, Carlsbad, CA) and sequence files were analyzed using Sequencher 4.10.1 (Gene Codes Corporation, Ann Arbor, MI).

**Table 1:** PCR primers and annealing temperature for each of the 16 exons

**Coding region**	**Forward sequence**	**Reverse sequence**	**Annealing temperature**
Exon 1	ggtgcctccggggctca	gaatgcgtccctcgcaagt	70.5°C
Exon 2	gagtcacccggttccatctacctt	ccagggacaccggcagc	70.5°C
Exon 3	tcttgtctttaatctgtccaatttcctaatctc	gcgcactaaaacaacagcga	63.5°C
Exon 4	tatccgtcttgaattgtcttatcttgttcctcatc	ccctcccagagaaacagagaactt	63.5°C
Exon 5	cagtgttgggatccttctttactaattc	cccggtgtcaacaagcttctaa	63.5°C
Exon 6	tttcttcctcatcagagctcaagt	tccaaagaacctaagagtctttctgagta	69.0°C
Exon 7	tcccaaagtgcagcttgtctaa	taatacacatttgtcctccacaccc	60.5°C
Exon 8	gtcctgacttggttgtgtcg	agacctttctttggaaaccctctaa	69.0°C
Exon 9	agtcttggtactttgtaaatgacacatct	agctgtgaggatgccagtt	69.0°C
Exon 10	aaatgtttcgttttgtttttaacttcattgt	ccagttgctgcaagtcagttga	67.5°C
Exon 11	tttcagctacatgttgtttgctggtcctatt	gctaggaggtcgaggcagcaaag	60.5°C
Exon 12	ttgccaagctgccacattt	gcatggcagttggagcaaag	63.5°C
Exon 13	cctcccctggtctcatcatttc	aagtcaaaggctgagtcacttgc	67.5°C
Exon 14	cttctttatctttggctctcaacactt	agagatcaccactgagctacc	63.5°C
Exon 15	cctactcttcattgtacttcaacct	tgagcttagagatgagccatgc	63.5°C
Exon 16.1	acaggtgtgcccttcctttcactaa	ccaccagcaacgtgatttctgcattt	60.5°C

Cycling conditions were: 95.0°C for two minutes, followed by 50 cycles of 94.0°C for 30 seconds and the specified annealing temperature for 30 seconds, followed by a single cycle of 94.0°C for 30 seconds and then 20.0°C for 30 seconds.

The A617T variant was investigated using TaqMan SNP genotyping assay C__32307311_10 (Life Technologies, Inc, Carlsbad, CA) using 10 ng of DNA; reactions were amplified in duplicate and genotypes (TT, TC or CC) were determined using the ABI PRISM^®^ 7000 Sequence Detection System software (Applied Biosystems, Foster City, CA).

## Results

### Clinicopathological characteristics

Of the 100 patients with ILCA, six had a self-reported history of gastric or stomach cancer in primary or secondary members of their family. None of the patients had a personal history of any type of cancer other than ILCA. The average age at diagnosis was 61.7 years (range 44–70 years). The majority of patients were Caucasian (83%), with early-stage (86%) and ER + HER2- (93%) lobular carcinomas. Thirty-two percent of the patients had a family history of breast cancer, defined as at least two primary or secondary relatives having been diagnosed with breast cancer at any age, while 20% of patients had bilateral breast cancer, similar to that seen in other ILCA populations [7]. Two of the patients, both diagnosed with stage IIIC luminal A ILCA have died of disease.

### Mutation results

No nonsense or splice site mutations were detected in any of the 100 genomic DNAs evaluated. A number of SNPs were detected in multiple individuals, including seven synonymous variants, two intronic variants and one variant in the 5’ UTR (Table 2). Four missense mutations were identified including private variant P825L, detected in a 62 year old white woman whose grandmother had stomach cancer, A592T in a 51 year-old white woman, A617T in two African Americans, both post-menopausal, and G879S in an 81 year-old white woman.

**Table 2:** Summary of variants in patients with ILCA

**SNP ID**	**SNP effect**	**Number of patients with variant (n = 100)**	**Ethnicity**^**a**^
c.71C > G	5’ UTR	3	W
345G > A	Synonymous	1	W
531 + 10G > C	Intronic	9	W
933C > G	Synonymous	1	AA
1680G > C	Synonymous	1	W
A592T	Missense	1	HS
A617T	Missense	1	AA
1896C > T	Synonymous	2	AA
2076 T > C	Synonymous	46	W, AA, HS, NA
1937-13 T > C	Intronic	6	W
95971C > T	Synonymous	6	W
P825L	Missense	1	W
G879S	Missense	1	W
101193C > A	Synonymous	2	AA

^a^Ethnicity groups are: AA = African American, HS = Hispanic, NA = Native American, W = White.

With the identification of A617T in two African American women, the variant was assessed in a panel of genomic DNA samples from 165 additional African American women with IDCA or mixed features of IDCA and ILCA. Ten women were carriers of the minor (T) allele and one additional patient, an African American woman diagnosed with with IDCA at age 71, was homozygous for the minor allele. The frequency of the T allele in this population was similar to that reported in dbSNP (http://www.ncbi.nlm.nih.gov/projects/SNP/snp_ref.cgi?rs=33935154) where it has been detected only in Sub-Saharan African and African American populations.

## Discussion

Identification of patients with hereditary predisposition to breast cancer is critical for optimum clinical management. Identification of carriers of causative mutations can assist in risk assessment and risk reduction, as well as in family counseling. In addition, hereditary cancers may have different phenotypes, requiring different treatment regimens. For example, patients with BRCA1 mutations are at risk for both bilateral breast disease as well as ovarian carcinoma; thus, women with BRCA1-associated cancers may be offered bilateral mastectomy and/or oophorectomy and may derive more benefit from PARP inhibitors compared to patients with sporadic breast cancer [12]. Patients with both hereditary and sporadic forms of ILCA may benefit from targeted therapeutics such as the use of FGFR1 inhibitors that block survival pathways and reverse tamoxifen resistance [13], however, those with hereditary ILCA may, as those with BRCA1 and BRCA2 mutations, be subjected to increased screening for bilateral disease, offered risk reduction strategies, as well encouraged to undergo prophylactic gastrectomy [14].

The frequency of potentially causative mutations in this study was low, with no instances of splice site or nonsense mutations detected. Only 5% (5/100) of patients had missense mutations. Under current guidelines in which genetic testing for BRCA1 and BRCA2 mutation is recommended for women with estimated probabilities of having a germline mutation ≥10% [15], genetic testing for CDH1 mutations in patients with ILCA would not be warranted. In addition, the clinical importance of the variants detected is unclear. To determine the pathogenicity of these variants, the pubmed database (http://www.ncbi.nlm.nih.gov/pubmed/) was searched for any citations that included these variants. In addition, the Catalog of Somatic Mutations in Cancer (COSMIC) database (http://cancer.sanger.ac.uk/cancergenome/projects/cosmic/) was searched. To our knowledge, variants P825L and G879S have not been documented as causative mutations in the literature, although a single instance of the P825L variant in a lung tumor was reported in the COSMIC database. Functional assessment of A592T suggests this variant is likely tolerable and non-pathogenic [16]. The A617T variant has been detected in a patient with endometrial cancer [17] and in two female African American patients with diffuse gastric cancer [18]. Transfection of CHO cells with CDH1 carrying the A617T variant resulted in an epithelial phenotype with extensive cell-to-cell contacts with membranous E-cadherin and β-catenin staining similar to that in wild-type cells [19]. Data from our study supports A617T as being a non-pathogenic variant; 10/165 African American women with IDCA and one with mixed IDCA and ILCA were carriers of the minor allele. In addition, one 71 year-old woman with IDCA was homozygous for the minor allele. The 6.7% minor allele frequency for A617T detected in our patient samples is identical to that reported in dbSNP for African American populations; thus this variant likely represents a non-pathogenic SNP specific to individuals with African ancestry. In addition, we performed a SIFT (**S**orting **I**ntolerant **F**rom **T**olerant) [20] analysis for the four non-synonymous variants. A592T, A617T and G879S were classified as tolerable variants, while P825L was predicted to be intolerable.

Our ability to detect mutations may be limited by the technical approach employed. Large deletions may not be detected by high-resolution melting analysis or direct sequencing of amplified products, however, the reported frequency of large-scale deletions in HDGC families is low (3.8%) [21], thus it is not likely that many of the ILCA patients have an undetected deletion. In addition, genomic DNA samples from family members were not collected, thus, it was not possible in this study to determine if the variants tracked with disease status. Finally, identification of patients with a family history of gastric cancer was based on self-reporting of cancer within first- and second-degree family members by the patient and ages at diagnosis were not provided. Of the 100 patients with ILCA, six had a single self-reported family member with stomach or gastric cancer, thus, the majority of these patients may not have a family history of HDGC.

## Conclusions

In our data of patients with ILCA unselected for age at diagnosis or family history of breast cancer, the mutation frequency was low – four missense mutations, only one of which (P825L) has been predicted to be pathogenic - in five individuals. These patients were recruited from a breast, rather than gastroenterology clinic, and thus the mutational frequency and spectrum differs based on presence or absence of a significant family history of HDGC. A previous study of 318 women with ILCA diagnosed <45 years or with a family history of breast cancer found no deletions or truncating mutations and of the six detected non-synonymous mutations, only four were found to be pathogenic, for a mutation frequency of 1.3% [22]. Together, these data suggest that the frequency of germline mutations in patients with ILCA is low, and screening for CDH1 mutations in this population is not warranted.

## Competing interests

The authors declare that they have no competing interests.

## Authors’ contributions

ALV performed the high-resolution melting and sequence analysis and reviewed the manuscript, SR performed the genotyping of the A617T variant and reviewed the manuscript, CDS oversaw collection of all patient samples and reviewed the manuscript, REE designed the project, performed data analysis and wrote the manuscript. All authors read and approved the final manuscript.

## References

[B1] Weigelt B Geyer FC Reis-Filho JS Histological types of breast cancer: how special are they? Mol Oncol 2010 4 192 208 10.1016/j.molonc.2010.04.004 20452298 PMC5527938

[B2] Borst MJ Ingold JA Metastatic patterns of invasive lobular versus invasive ductal carcinoma of the breast Surgery 1993 114 637 641 8211676

[B3] Martinez V Azzopardi JG Invasive lobular carcinoma of the breast: incidence and variants Histopathology 1979 3 467 488 10.1111/j.1365-2559.1979.tb03029.x 229072

[B4] Fisher ER Gregorio RM Fisher B Redmond C Vellios F Sommers SC The pathology of invasive breast cancer: a syllabus derived from findings of the National Surgical Adjuvant Breast Project (protocol no. 4) Cancer 1975 36 1 85 10.1002/1097-0142(197507)36:1<1::AID-CNCR2820360102>3.0.CO;2-4 173455

[B5] Krecke KN Gisvold JJ Invasive lobular carcinoma of the breast: mammographic findings and extent of disease at diagnosis in 184 patients AJR Am J Roentgenol 1993 161 957 960 10.2214/ajr.161.5.8273634 8273634

[B6] Lesser ML Rosen PP Kinne DW Multicentricity and bilaterality in invasive breast carcinoma Surgery 1982 91 234 240 6277027

[B7] Arpino G Bardou VJ Clark GM Elledge RM Infiltrating lobular carcinoma of the breast: tumor characteristics and clinical outcome Breast Cancer Res 2004 6 R149 R156 10.1186/bcr767 15084238 PMC400666

[B8] Paredes J Figueiredo J Albergaria A Oliverira P Carvalho J Ribeiro AS Caldeira J Costa AM Simoes-Correia J Oliveira MJ Pinheiro H Pinho SS Mateus R Reis CA Leite M Ferdandes MS Schmitt F Carneiro F Figueiredo C Oiveira C Seruca R Epithelial E- and P-cadherins: role and clinical significance in cancer Biochem Biophys Res Commun 1826 2012 297 311 10.1016/j.bbcan.2012.05.002 22613680

[B9] Pharoah PD Guilford P Caldas C Incidence of gastric cancer and breast cancer in CDH1 (E-cadherin) mutation carriers from hereditary diffuse gastric cancer families Gastroenterology 2001 121 1348 1353 10.1053/gast.2001.29611 11729114

[B10] Fitzgerald RC Hardwick R Huntsman D Cameiro F Guilford P Blair V Chung DC Norton J Ragunath K Van Krieken JH Dwerryhouse S Caldas C International Gastric Cancer Linkage Consortium Hereditary diffuse gastric cancer: updated consensus guidelines for clinical management and directions for future research J Med Genet 2010 47 436 444 10.1136/jmg.2009.074237 20591882 PMC2991043

[B11] American Joint Committee on Cancer AJCC Cancer Staging Manual 2010 7 New York: Springer

[B12] Silver DP PARP Inhibition in Triple Negative Breast Cancer 2011 8 November, 2011

[B13] Sikora MJ Cooper KL Bahreini A Luthra S Wang G Chandran UR Davidson NE Dabbs DJ Welm AL Oesterreich S Invasive lobular carcinoma cell lines are characterized by unique estrogen-mediated gene expression patterns and altered tamoxifen response Cancer Res 2014 74 1463 1474 10.1158/0008-5472.CAN-13-2779 24425047 PMC3955299

[B14] Kluijt I Sijmons RH Hoogerbrugge N Plukker JT de Jong D Van Krieken JH van Hillegersberg RL Ligtenberg M Bleiker E Cats A Dutch Working Group on Hereditary Gastric Cance Familial gastric cancer: guidelines for diagnosis, treatment and periodic surveillance Fam Cancer 2012 11 363 369 10.1007/s10689-012-9521-y 22388873

[B15] Statement of the American Society of Clinical Oncology: genetic testing for cancer susceptibility J Clin Oncol 1996 14 1730 1736 8622094 10.1200/JCO.1996.14.5.1730

[B16] Garziera M Canzoneiri V Cannizzaro R Geremia S Caggiari L De Zorzi M Maiero S Orzes E Perin T Zanussi S de Paoli P De Re V Identification and characterization of CDH1 germline variants in sporadic gastric cancer patients and in individuals at risk of gastric cancer PloS ONE 2013 8 e77035 10.1371/journal.pone.0077035 24204729 PMC3812172

[B17] Risinger JI Berchuck A Kohler MF Boyd J Mutations of the E-cadherin gene in human gynecologic cancers Nat Genet 2014 7 98 102 8075649 10.1038/ng0594-98

[B18] Suriano G Oliveira C Ferreira P Machado JC Bordin MC De Wever O Bruyneel EA Moguilevsky N Grehan N Porter TR Richards FM Hruban RH Roviello F Huntsman D Mareel M Carneiro F Caldas C Seruca R Identification of CDH1 germline missense mutations associated with functional inactivation of the E-cadherin protein in young gastric cancer probands Hum Mol Genet 2014 12 575 582 12588804 10.1093/hmg/ddg048

[B19] Suriano G Oliveira MJ Huntsman D Mateus AR Ferreira P Casares F Oliveira C Cameiro F Machado JC E-cadherin germline missense mutations and cell phenotype: evidence for the independence of cell invasion on the motile capabilities of the cells Hum Mol Genet 2003 12 3007 3016 10.1093/hmg/ddg316 14500541

[B20] Ng PC Henikoff S SIFT: predicting amino acid changes that affect protein function Nucleic Acids Res 2003 31 3812 3814 10.1093/nar/gkg509 12824425 PMC168916

[B21] Oliveira C Senz J Kaurah P Pihheiro H Sanges R Haegert A Corso G Schouten J Fitzgerald R Vogelsang H Keller G Dwerryhouse S Grimmer D Chin SF Yang HK Jackson CE Seruca R Roviello F Stupka E Caldas C Huntsman D Germline CDH1 deletions in hereditary diffuse gastric cancer families Hum Mol Genet 2009 18 1545 1555 10.1093/hmg/ddp046 19168852 PMC2667284

[B22] Schrader KA Masciari S Boyd N Salamanca C Senz J Saunders DN Yorida E Maines-Bandiera S Kaurah P Tung N Robson ME Ryan PD Olopade OI Domchek SM Ford J Issacs C Brown P Balmana J Razzak AR Miron P Coffey K Terry MB John EM Andrulis IL Knight JA O’Malley FP Daly M Bender P kConFab Moore R Germline mutations in CDH1 are infrequent in women with early-onset or familial lobular breast cancers J Med Genet 2011 48 64 68 10.1136/jmg.2010.079814 20921021 PMC3003879

